# Legislative Enforcement of Nonconsensual Determination of Neurological (Brain) Death in Muslim Patients: A Violation of Religious Rights

**DOI:** 10.1007/s10943-017-0512-z

**Published:** 2017-10-24

**Authors:** Mohamed Y. Rady, Joseph L. Verheijde

**Affiliations:** 1 Mayo Clinic College of Medicine and Science and Department of Critical Care Medicine, Mayo Clinic Hospital, 5777 East Mayo Blvd, Phoenix, AZ 85054 USA; 2 Mayo Clinic College of Medicine and Science and Department of Physical Medicine and Rehabilitation, Mayo Clinic, 13400 E Shea Blvd, Scottsdale, AZ 85259 USA

**Keywords:** Brain death, Organ donation, End-of-life care, Islam, Neuroscience, Disorders of consciousness, Religion

## Abstract

Death is defined in the Quran with a single criterion of irreversible separation of the *ruh* (soul) from the body. The Quran is a revelation from God to man, and the primary source of Islamic knowledge. The secular concept of death by neurological criteria, or brain death, is at odds with the Quranic definition of death. The validity of this secular concept has been contested scientifically and philosophically. To legitimize brain death for the purpose of organ donation and transplantation in Muslim communities, Chamsi-Pasha and Albar (concurring with the US President’s Council on Bioethics) have argued that irreversible loss of capacity for consciousness and breathing (apneic coma) in brain death defines true death in accordance with Islamic sources. They have postulated that the absence of *nafs* (personhood) *and nafas* (breath) in apneic coma constitutes true death because of departure of the soul (*ruh*) from the body. They have also asserted that general anesthesia is routine in brain death before surgical procurement. Their argument is open to criticism because: (1) the *ruh* is described as the essence of life, whereas the *nafs* and *nafas* are merely human attributes; (2) unlike true death, the *ruh* is still present even with absent *nafs and nafas* in apneic coma; and (3) the routine use of general anesthesia indicates the potential harm to brain-dead donors from surgical procurement. Postmortem general anesthesia is not required for autopsy. Therefore, the conclusion must be that legislative enforcement of nonconsensual determination of neurological (brain) death and termination of life-support and medical treatment violates the religious rights of observant Muslims.

## Introduction

Brain death, or death determination by neurological criteria, remains conceptually controversial on scientific, philosophical, and theological grounds (Joffe 2009; Nature 2009; The Lancet 2011; Wahlster et al. 2015). The Quran is a revelation from God to man, and the primary source of Islamic knowledge. Death is defined in the Quran as the irreversible separation of the *ruh* (soul) from the body, and this separation is associated with biological disintegration (Table 1). The onset of disintegration is confirmed by the loss of thermodynamic entropy in biological systems (Nahle 2009). To advance organ donation and transplantation in Western medical practice, a customized secular definition of death was formulated (Rady and Verheijde 2013). This secular definition of death equates irreversible loss of capacity for consciousness and breathing (apneic coma) with death despite a person’s normal heart function and circulation. This particular definition was reviewed and again endorsed in the US President’s Council on Bioethics White paper, as well as the UK code of practice for the diagnosis of death (The President’s Council on Bioethics 2008; The Academy of Medical Royal Colleges 2008). The same definition was also endorsed by the World Health Organization and outlined in the international guidelines for death determination (Shemie et al. 2014). The underlying premise for accepting death by neurological criteria was that the capacity for consciousness and breathing together form the two fundamental human attributes to sustain life. Absence of these capacities, it was argued, equated to human death.

**Table 1:** Description of the phenomenon of death in the Quran

The characteristics of the phenomenon of death	The criteria in the determination of death
• God created the phenomenon of death • The phenomenon of death is universal and singular • The definition of death is uniform and constant across generations and geography • The determination of death requires *yaqin* (absolute certainty) • The process of dying must be distinguished from the state of death	• Ruh (soul) has separated irreversibly from the body • Ruh is present in the body as long as the brain and the heart retain capacity for recovery of function • Ceased vital functions can no longer be reversed regardless of any external intervention (absolute irreversibility) • The biological criterion (disintegration) confirms death

The Quran is a revelation from God to man and the primary source of Islamic knowledge

Chamsi-Pasha and Albar (2017) have endorsed the US President’s Council on Bioethics philosophical defense of the concept of brain death. They have argued that death determination by neurological criteria (brain death) exclusively, rather than by traditional cardiorespiratory criteria, is permissible based on Islamic sources. In support of their claim, the authors have postulated that the absence of *nafs* (personhood) and *nafas* (breath) in apneic coma (the core diagnosis of brain death) confirms the departure of the soul from the body. This novel interpretation seeks to align the Quranic definition of death with the secular construct of brain death. Such alignment is essential to sanction the surgical procurement of transplantable organs from brain-dead heart-beating donors in Muslim communities. Although Chamsi-Pasha and Albar (2017) have argued that brain death is true death, they have also asserted, contrary to their claim of true death, that general anesthesia is routinely administered in brain-dead donors before surgical procurement of transplantable organs. They have called upon governments to legislatively enforce nonconsensual determination of neurological (brain) death and immediate termination of medical care, including life-support treatment, in Muslim patients who are not organ donors, in order to conserve healthcare resources.

In this commentary, firstly, a definition of death equating apneic coma with irreversible *ruh* separation from the body will be contested because it clashes with the Quranic definition of death. It will be argued that the *ruh* is distinct from the *nafs* and *nafas*. The *ruh* is the essence of life, while the *nafs* and *nafas* are merely human attributes. Therefore, unlike in true death, the *ruh* continues to be linked to the body of persons declared brain-dead, even in the absence of *nafs* and *nafas*. Secondly, a challenge will be posed to the claim that general anesthesia is routinely administered in brain-dead donors before organ procurement. It will be argued here that this implicitly underscores the notion that brain-dead donors are believed to be living human beings and not cadavers. True human cadavers need no general anesthesia for postmortem organ procurement. Thirdly, the authors will examine the claim that the medical care of brain-dead nondonors is more burdensome on healthcare resources than that of brain-dead organ donors. Finally, based on these considerations the authors conclude that legislative enforcement of nonconsensual determination of brain death and termination of life-support and medical treatment in nondonors violates the religious rights of observant Muslims.

## The Quranic Definition of Life and Death

Chamsi-Pasha and Albar (2017) used *al*-*nafs* ( ) and *al*-*ruh* ( ) interchangeably to define the transition from life (*al*-*hayata* = ) to death (*al*-*mawta* = ). The *nafs* is differentiated from the *ruh* in the Quran. The *nafs* describes human attributes and personhood, while the *ruh* is the essence of life in the human body. Life begins with *ruh* ensoulment and ends with its separation from the body:

Then He made his offspring from semen of despised water (8) Then He fashioned him in due proportion, and breathed into him ruh-ihi (the soul created by Allah [God] for that person); and He gave you hearing (ears), sight (eyes) and hearts. Little is the thanks you give (9) (The Quran Chapter 32 verses 8–9).

The irreversible separation of the *ruh* from the body is death. There is only one physical death in the Quran and that is death of the whole human being. God created this singular phenomenon of death, and his definition does not change with time or place:

Blessed be He in Whose Hand is the dominion; and He is Able to do all things. (1) Who has created al-mawta (death) and al-hayata (life) that He may test you which of you is best in deed. And He is the All-Mighty, the Oft-Forgiving (2) (The Quran Chapter 67 verses 1–2).

**Fig. 1:**
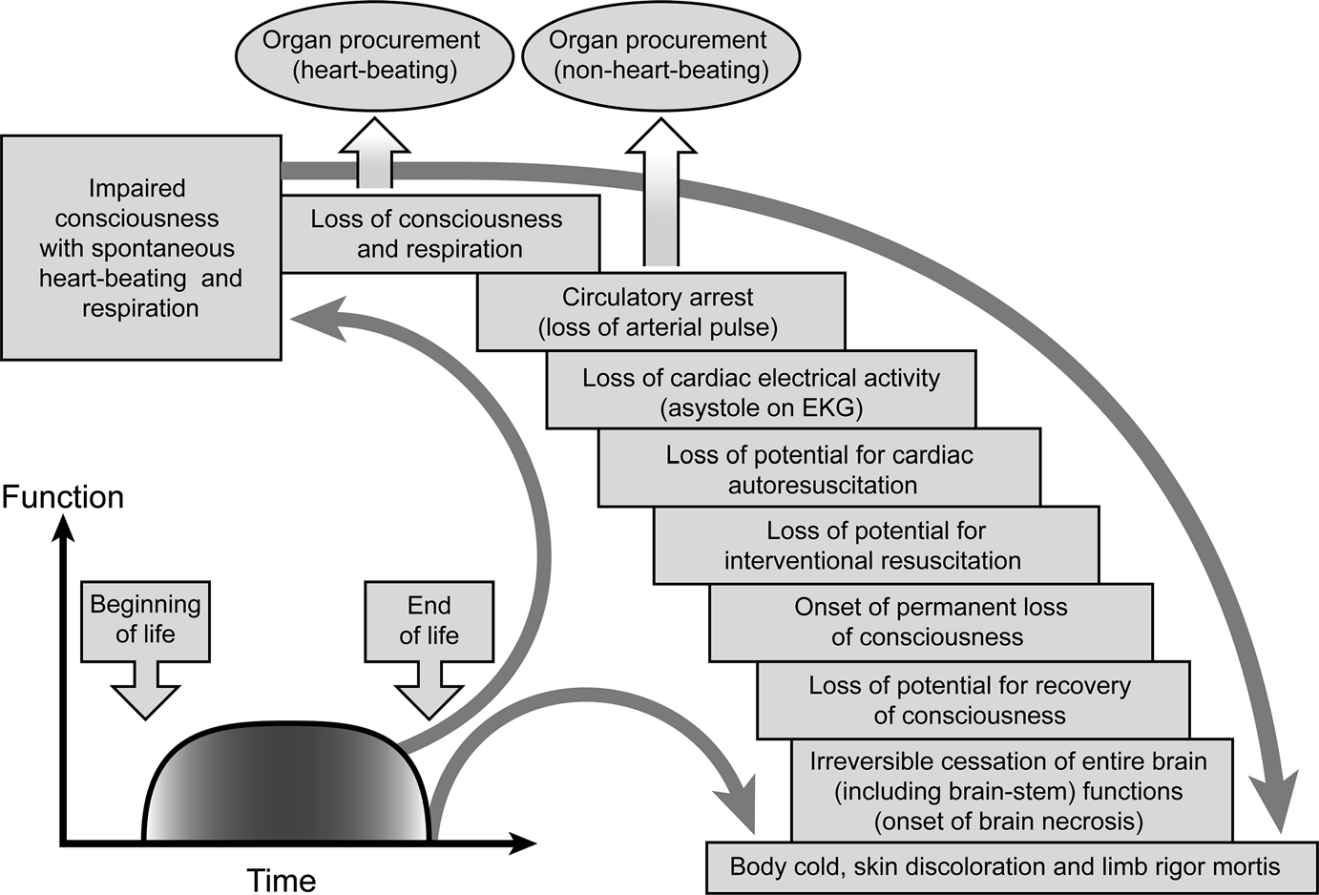
“Human death is a singular phenomenon. The dying process occurs in stages over time. There is a gradual loss of capacity for somatic integration of the whole body because of an irreversible cessation of all vital and biological functions including circulation, respiration (controlled by the brainstem), and consciousness. The irreversibility of cessation of circulatory and respiratory functions is interlinked to the onset of whole brain necrosis. The loss of capacity for consciousness is irreversible when the necrosis of the whole brain, including the brainstem, is complete”. “There is no accurate clinical test to ascertain the absence of self and/or environmental awareness in unresponsive patients following severe brain injuries. Arbitrary neurological and circulatory criteria redefining human death enable heart-beating and non-heart-beating procurement of transplantable organs, respectively. Scientifically flawed criteria of death can harm donors because procurement procedures are performed without general anaesthesia” (Rady and Verheijde 2014). Figure reproduced from source (Verheijde et al. 2009), under the terms of the Creative Commons Attribution License (http://creativecommons.org/licenses/by/3.0/)

The Quran differentiates the process of dying from the state of true death (Table 1). The time of death is when the biological events during the process of dying are completed (Fig. 1). Therefore, the phenomenon of death is only discoverable through scientific inquiry and should not be redefined or reconstructed otherwise to better accommodate special interests such as organ donation and transplantation (Nair-Collins 2015). The core of the diagnosis of death by neurological criteria (brain death) consists of irreversible loss of capacity for consciousness and breathing; this construct of death enables the surgical procurement of transplantable vital organs from heart-beating donors. The US President’s Council on Bioethics (2008) has equated irreversible cessation of capacity for consciousness and breathing with the death of a living organism as a *philosophical* explanation of the concept of brain death. The Council has considered the combined capacity for consciousness and spontaneous breathing to be a fundamental attribute of a living organism, as it allows the organism to commerce with its external environment. It has been proposed and accepted that the absence of consciousness and spontaneous breathing indicates the death of the whole organism despite preservation of other biological functions and somatic integration of the whole body. However, in personal statements, Alfonso Gómez-Lobo (p. 95) and Edmund D. Pellegrino (p. 107), both members of the Council, expressed opposition to this concept of death (The President’s Council on Bioethics 2008). Shewmon (2009) probably best characterized the report’s contribution to the resolution of conceptual problems surrounding the notion of brain death:

Unfortunately*, the new solution does not put the problem to rest, but the humility with which the council discusses its own position and the honesty with which it confronts the consequences of being wrong* alone make this report a very commendable document. [Emphasis added].

Chamsi-Pasha and Albar have accepted the US President’s Council’s philosophical formulation in defense of the concept of brain death at its face value and without further justification for their endorsement:

Human death involves the irreversible loss of the capacity for consciousness, combined with the irreversible loss of the capacity to breathe… The US President’s Council on Bioethics proposed a new unifying concept of death in 2008. The Council reiterated its support for a whole brain [death] formulation (Chamsi-Pasha and Albar 2017).

In addition, Chamsi-Pasha and Albar have attempted to equate irreversible apneic coma with the separation of the *soul* from the body. The terms deep coma or coma dépassé (“a state beyond coma”) were used when brain death was initially equated to human death in 1959 (Mollaret and Goulon 1959). However, 50 years ago the fields of neurology and neuroscience research were in their infancy. Since then, empirical breakthroughs in neuroscience research have dispelled many old dogmas in the clinical practice of neurology (Gross 2000). Nevertheless, the outdated dogmatic understanding of human consciousness continues to be entrenched in US and UK clinical practice guidelines for brain death determination (Peterson et al. 2014; Rady and Verheijde 2016a, b; Choong and Rady 2016). Advances in neuroscience have rendered the term *deep coma* obsolete and have replaced it with the more accurate term *disorders of consciousness* (Giacino et al. 2014; Peterson et al. 2015; Naci et al. 2017). This term represents a spectrum of phenotypes that arises from the neuropathophysiology of consciousness involving receptivity and responsiveness (Rady and Verheijde 2017). Neuroscience research has also refuted contemporary understanding of *irreversibility* of cessation of brain functions and, more specifically, the capacity for consciousness: “[p]ortions of the post-mortem human brain may retain latent capacities to respond with potential life-like and virtual properties” similar to a living human brain (Rouleau et al. 2016).

Chamsi-Pasha and Albar have equated *al*-*mawta,* or death in brain death, with *al*-*wafat* ( ), a state during sleep (*al*-*nuwma* = ), and have posited equivalence of these terms, arguing that the soul *has departed* from the body in both states:

It is also mentioned in the Quran that sleep is similar in a way to death. Body is a home for the soul. The soul departs at sleep and returns when it is time to get up. People move from one state, with its governing laws, to a different state, with completely different laws each day without knowing or thinking about it (Chamsi-Pasha and Albar 2017).

They cited the following Quranic verse to substantiate their claim of soul departure in both brain death and sleep:

It is Allah [God] Who takes away alanfusa (the souls) at the time of their death, and those that die not during their sleep. He keeps those (souls) for which He has ordained death and sends the rest for a term appointed. Verily, in this are signs for a people who think deeply. (The Quran Chapter 39 verse 42).

*Al*-*anfusa* is the plural of *al*-*nafs*. Chamsi-Pasha and Albar have interpreted the *al*-*anfusa* in the Quranic verse (*yatawaffa alanfusa*— ) to be the same as the *ruh,* suggesting that if the *nafs* is taken away during sleep (*fee manamiha*— ), then the *ruh* would also be separated from the body in an unconscious and a brain-dead person. This interpretation and inferential conclusion is open to serious challenges. Firstly, sleep and brain death are different states and have distinguishable neurophysiological features. Neuroscience has described specific patterns of electrical activity of the human brain on electroencephalography that are characteristic of the different stages of sleep (Aboalayon et al. 2016; Ducharme-Crevier et al. 2017). Disorders of consciousness, including brain death, have different electroencephalographic patterns distinguishable from that found during sleep (Landsness et al. 2011; Fyntanidou et al. 2012). Secondly, if the interpretation of Chamsi-Pasha and Albar is correct, a sleeping person ought to be presumed dead and could qualify as a brain-dead donor. Thirdly, Chamsi-Pasha and Albar have overlooked that the Quran has distinguished the state of sleep from death based on the relationship of the *ruh* (rather than the *nafs*) with the body. The *ruh* is still linked to the body during sleep. The *nafs* is taken away in *al*-*wafat* during sleep and returned back on waking up (*wayursilu alokhra ila ajalin musamman*— ). In death, the *ruh* and the *nafs* are taken away and are no longer linked to the body (*fayumsiku allatee qada alayha almawta*— ).

It is He Who takes your souls by night (when you are asleep), and has knowledge of all that you have done by day, then He raises (wakes) you up again that a term appointed (your life period) be fulfilled, then (in the end) unto Him will be your return. Then He will inform you of that which you used to do. (60) He is the Irresistible, (Supreme) over His slaves, and He sends guardians (angels guarding and writing all of one’s good and bad deeds) over you, until when death approaches one of you, Our Messengers (angel of death and his assistants) take his soul, and they never neglect their duty (61) (The Quran Chapter 6 verses 60–61).

Finally, the aforementioned Quranic verses verify that *al*-*wafat* is different from *al*-*mawta* (death). *Al*-*wafat* can be a reversible state, such as during sleep; however, *al*-*mawta* is an irreversible state. The irreversible separation of the *ruh* determines irreversibility of *al*-*mawta* and confirms man’s helplessness to reverse true death (Table 1). To summarize, Chamsi-Pasha and Albar have not provided conclusive evidence that both the *ruh* and *nafs* have irreversibly departed from the body in either the state of coma or of brain death, a necessary condition for compliance with the Quranic definition of death.

The *nafs* describes human attributes and personhood in the Quran. Because *nafs* is similar to *nafas* (breath), Chamsi-Pasha and Albar have asserted that the simultaneous absence of consciousness (*nafs*) and breathing (*nafas*) equates with separation of the *ruh* from the body and thus constitutes death. If this proposed definition of death is to be accepted, then all patients who are dependent on mechanical ventilation for breathing and who may be under general anesthesia or asleep are to be considered dead from an Islamic perspective. It further implies that a nine-month full-gestation fetus ought also to be considered dead until the moment the fetus is born and breathing.

An alternative biological definition of death based on cessation of homeostasis and integration of cells, tissue, organs, and the whole body (biological disintegration) has been proposed that is consistent with irreversible separation of the *ruh* from the body (Rady and Verheijde 2016c). Chamsi-Pasha and Albar (2017) have critiqued this biological definition of death as follows:

Rady and Verheijde (2016c) argue that the residual functions of the central nervous system, homeostasis, and somatic integration of the whole body persist in brain death. They claim that death is biologically defined in the Quran by disintegration as emphasized in several Quranic verses…*This definition is incorrect since there is a prolonged period elapsing between the moment of death; i.e., departure of soul from the body, and the* *disintegration of a corpse into dust and bones and nobody would wait until the bones of the dead become dust”* [Emphasis added].

Chamsi-Pasha and Albar’s interpretation of the biological definition of death is incorrect because the disintegration of the body into bone and dust is not a requirement in the proposed definition. Instead, the pathophysiological events that are associated with the *process of dying* (Fig. 1) should be completed before death is declared (Verheijde et al. 2009). The final outcome of death is reached when the biological systems are no longer integrated and cease to generate cellular energy:

The Quran explicitly differentiates between the process of dying and death, the latter being the final outcome of that process… *The onset of disintegration begins after the completion of the dying process.* *In thermodynamic and biologic terms, disintegration is associated with an irreversible loss of thermodynamic entropy (internal energy generation) and the loss of homeostasis and cellular, tissue, organ, and whole body integration…Once cellular energy generation ceases, the biologic system begins disintegration and declines rapidly into thermodynamic equilibrium with the external environment…* In contrast, the process of dying is a gradual process over time during which the ceased vital functions are reversible… (Rady and Verheijde 2016c) [Emphasis added].

The cessation of vital functions should be irreversible in death (Table 1); therefore, a biological and thermodynamic definition of death is better aligned with the Quranic characterization of death than the definition proposed by Chamsi-Pasha and Albar which is focused on loss of a specific subset of human attributes (consciousness and breathing) while other attributes of a living human being are still present.

## General Anesthesia for Brain-Dead Organ Donors

General anesthesia is not required for performing autopsy on human cadavers. However, brain-dead donors do show both physical and physiological reactions to the surgical procedure of organ procurement when performed with no general anesthesia (Young and Matta 2000). Donors can display limb movements in response to the surgical procedure and those are routinely suppressed via administration of neuromuscular-blocking drugs that induce skeletal muscle paralysis (Anderson et al. 2015). Whenever *general anesthesia* is administered, hypnotic and analgesic drugs are required to eliminate both awareness and pain of the surgical procedure. In brain-dead donors, *only* neuromuscular-blocking drugs are given to induce skeletal muscle paralysis and without concurrent administration of analgesic or hypnotic drugs to alleviate potential nociception and/or awareness, respectively (Rady and Verheijde 2013). Administration of *general anesthesia* to brain-dead donors during surgical procurement is not described in most organ procurement policies and is de facto not a universal standard practice as suggested by Chamsi-Pasha and Albar:

In fact, *most surgeons require general anesthesia to procure organs from brain*-*dead individuals* to avoid spinal reflexes. (Chamsi-Pasha and Albar 2017) [Emphasis added]

Donors can potentially suffer during organ procurement if general anesthesia is not universally administered. The need for routinely administering general anesthesia in organ donors who have been declared dead, however, raises two fundamental questions: (1) what differentiates the need for general anesthesia in brain-dead donors undergoing surgical procurement from the nonuse of postmortem anesthesia in cadaveric autopsy, and (2) when consent to donation is obtained, are families or next-of-kin informed that their relatives declared brain-dead require general anesthesia during surgical organ procurement so as not to inflict harm on them?

## Legislative Enforcement of Brain Death Determination in Muslim Patients

Chamsi-Pasha and Albar (2017) have stated “…there are still some uncertainties about the concept of brain death among some Muslim scholars.” They have argued that “consensus on brain death is long overdue” without addressing persistent neuroscience, medical practice, or philosophical problems. The criteria for clinical determination of brain death still lack scientific validation almost 50 years since the original inception of this concept of death (Varelas 2014; Smith and Citerio 2017). Both the US and the UK clinical practice guidelines for brain death determination have been scientifically challenged (Choong and Rady 2016; Rady and Verheijde 2016a). Contemporary neuroscientists have also challenged the scientific foundation and validation of contemporary criteria in brain death (Peterson et al. 2014). Indeed, neuroscience research in disorders of consciousness has made several breakthrough advances that confirmed the complexity of clinical assessment of the capacity for consciousness in the severely injured brain (Rady and Verheijde 2016b). Latent and covert conscious awareness can persist following severe brain injuries (Fernández-Espejo et al. 2015; Edlow et al. 2017). However, the precise timeline of potential recovery of consciousness remains unknown. Currently, there are no reliable clinical tools or tests that can accurately ascertain the irreversible loss of capacity for consciousness in the severely injured brain. Clinical testing for absent motor reflexes of the cranial nerves (including respiratory centers) located in the brainstem *does not* verify or ascertain irreversible cessation of capacity for consciousness. The reliance on absent motor or behavioral responses to stimuli to confirm absence of consciousness and of higher integrative cerebral functions is prone to errors. Consciousness has been detected in the absence of motor and behavioral responses in a state described as cognitive motor dissociation following severe brain injuries (Schiff 2015, 2017). The presence of scientific doubt about the validity of brain death determination is not permitted in Islam because the Quran characterizes death with *yaqin* (absolute certainty) (Table 1) and mandates certainty in its determination, i.e., a singular event determined with an absolute certainty at a specific time: “And worship your Lord until there comes unto you the certainty (i.e., death)” (The Quran Chapter 15 verse 99).

The conception of brain death is grounded in a secular philosophical interpretation of the value of human life on the basis of personhood (Choong and Rady 2016). Contemporary medical criteria for death determination in end-of-life organ donation (Fig. 1) permit surgical procurement before true death of donors and is *de facto* a concealed form of physician-assisted death (Verheijde et al. 2009). Physician-assisted death is forbidden in Islam. As a consequence, there has been a global upsurge in the medical, legal, and religious challenges to the concept of brain death (Lewis and Greer 2017). The transplant community has perceived this global increase in brain death controversies as a threat to the medical authority and power to declare death by neurological criteria only. This is certain to impede the organ donation and transplantation practice, and transplantation advocates have therefore called for additional governmental support through legislative enforcement of nonconsensual determination of brain death (Lewis and Pope 2017; Lewis and Greer 2017; Lewis 2017). Legislative enforcement of nonconsensual brain death determination will harm patients and violate their constitutional rights (Choong and Rady 2016; Rady et al. 2017). Chamsi-Pasha and Albar (2017) have also urged for legislative enforcement of nonconsensual determination of brain death and immediate termination of life-support and medical treatment in Muslim patients who are not organ donors on the grounds of futility of treatment and avoidance of wastefulness of limited healthcare resources. In contrast, life-support and medical treatment is continued in brain-dead donors until surgical procurement of transplantable organs for the “benefit obtained and the new life given to the recipient” (Chamsi-Pasha and Albar 2017). However, others may perceive the continuation or termination of life-support and medical treatment on the basis of an individual’s donor status as discriminatory medical practice.

Chamsi-Pasha and Albar (2017) have claimed that the medical care of Muslim brain-dead nondonors represents financial wastefulness of healthcare resources because these individuals “suffer miserable lives and consume significant resources” while having no options for “meaningful survival.” Other commentators have made similar arguments to justify an expeditious nonconsensual termination of life-support and medical treatment in brain-dead nondonors who have religious objection to brain death determination (Lewis et al. 2016, 2017; Lewis and Pope 2017). Chamsi-Pasha and Albar’s position invites a factual analysis of whether continued medical care of brain-dead nondonors is indeed more financially burdensome than that of brain-dead donors. First, it should be noted that brain-dead nondonors do not require intensive care unit (ICU) occupancy after initial medical stabilization of the acute phase of their brain injuries (Shewmon 1998; Muramoto 2016). Their average daily cost of ICU care is estimated at 5000 US dollars per patient (Khandelwal et al. 2016). Second, the long-term medical care of brain-dead nondonors with mechanical ventilation (through a tracheostomy) and enteral nutrition (through a gastrostomy) does not require ICU occupancy (Brown 2017). They can be discharged to nursing facilities or their homes in jurisdictions that accommodate religious objection to death determination by neurological criteria (Muramoto 2016; Brown 2017). In contrast, brain-dead donors do require ICU occupancy and management until surgical procurement (Kotloff et al. 2015). In 2014, approximately 6791 eligible US donors were managed in the ICU (Israni et al. 2016). In the same year, there were 27,567 transplant recipients of single- or multiple-solid organs with the sum of total healthcare charges in excess of 15 billion US dollars (Hanson and Bentley 2014). The total costs per transplant recipient included the charges for organ procurement in addition to the fees for the first 6 months of subsequent medical care (Table 2). The sum of procurement charges was in excess of 2 billion US dollars (Hanson and Bentley 2014). The healthcare expenditures related to the procurement of a brain-dead donor were between 377,300 and 676,600 US dollars. Although the charges are, in part, reflective of the US healthcare system, it highlights the fact that caring for brain-dead organ donors can strain limited ICU resources, and their care is no less costly than the long-term care provided to brain-dead nondonors. Additionally, the costs associated with end-of-life organ procurement are expensed out to transplant recipients. All of this can result in economic hardship for healthcare systems in countries with limited financial resources. Some commentators have also criticized the lack of financial transparency in organ procurement practices (Saari and Cooper 2017).

**Table 2:** The 2014 US procurement and transplant charges

Type of organ(s) transplant	Total estimated number of transplants (*n*)	Estimated billed charges per transplant^a^ ($)	Total billed charges^c^ ($)	Procurement charges per transplant^b^ ($)	Total procurement charges^d^ ($)
Heart	2320	1,242,200	2,881,904,000	97,200	225,504,000
Intestine	51	1,547,200	78,907,200	92,100	4,697,100
Kidney	15,978	334,300	5,341,445,400	84,400	1,348,543,200
Liver	5723	739,100	4,229,869,300	95,000	543,685,000
Lung-single	681	785,000	534,585,000	90,200	61,426,200
Lung-double	1220	1,037,700	1,265,994,000	129,700	158,234,000
Pancreas	149	317,500	47,307,500	93,800	13,976,200
Heart–lung	29	2,313,600	67,094,400	168,700	4,892,300
Intestine and other organs	49	1,844,700	90,390,300	236,400	11,583,600
Kidney–heart	85	1,840,300	156,425,500	136,000	11,560,000
Kidney–pancreas	773	558,600	431,797,800	123,300	95,310,900
Liver–kidney	471	1,190,300	560,631,300	161,500	76,066,500
Other–multi-organ	38	1,620,800	61,590,400	177,600	6,748,800
Total sum	27,567		15,747,942,100		2,562,227,800

Data source is the 2014 US organ and tissue transplant cost estimates and discussion report (Hanson and Bentley 2014)

Sum of procurement charges for a potential brain-dead donor = heart + lung-double + 2 × kidney + Liver + pancreas + intestine = [97,200 + 129,700 + (2 × 84,400) + 95,000 + 93,800 + 92,100] = $676,600

^a^Estimated billed charges per transplant: The charges included 30-day pre-transplant medical services, hospital transplant admission, organ procurement, transplant physician service, 180 days post-transplant follow-up, and outpatient immunosuppressants and other drugs associated with the transplant patient

^b^Procurement charges per transplant: The charges for organ or tissue recovery services included retrieval, preservation, transportation, and other acquisition costs. The charges included donor management in the intensive care unit

^c^Total billed charges = number of transplant recipients × estimated billed charges per transplant

^d^Total procurement charges = number of transplant recipients × procurement charges per transplant

## Conclusions

Chamsi-Pasha and Albar have argued that the *nafs* (personhood) and *nafas* (breath) are absent in apneic coma (the core diagnosis of brain death) and indicate the departure of *ruh* (soul) from the body. This secular definition of death is incongruent with the Quran. The Quran distinguishes the *ruh* from the *nafs* and *nafas.* The *ruh* is the essence of life in the body. The *nafs* and *nafas* are human attributes. The irreversible separation of the *ruh* from the body defines death. Unlike in true death, the *ruh* continues to be linked to the body in brain death. The requirement of general anesthesia before organ procurement confirms that brain-dead donors are still living beings who can suffer, and not merely human cadavers. True human cadavers do not need the administration of general anesthesia for postmortem autopsy or organ procurement. Neuroscience has questioned the validity of the concept of brain death and the clinical practice guidelines for determining neurological death, which in turn should caution all those who are attempting to align the concept of brain death with the Quranic definition of death. An alignment of brain death with the Quranic definition of death appears unattainable because: (1) robust, high-quality scientific evidence of the validity of the concept of death by neurological criteria is absent, and (2) philosophical consensus on this concept’s validity has not been established. As a consequence, legislative enforcement of nonconsensual determination of brain death and termination of life-support and medical treatment violates the religious rights of observant Muslims.
